# 3D transvaginal ultrasound diagnosis of uterine septa according to different classifications: are there other measurements that correlate to reproductive outcome in small indentation length?

**DOI:** 10.52054/FVVO.14.2.025

**Published:** 2022-07-01

**Authors:** C Russo, F Conway, T Siciliano, A Selntigia, F Giuseppe Martire, G Soreca, C Ticconi, C Exacoustos

**Affiliations:** Department of Surgical Sciences, Obstetrics and Gynecology Clinic, University of Rome “Tor Vergata”, Rome, Italy

**Keywords:** uterine congenital anomalies, septate uterus, 3D transvaginal ultrasound, infertility, recurrent miscarriage

## Abstract

**Background:**

High discrepancy between current classifications was observed in the definition of uterine septa, especially for indentation lengths >5 <10mm.

**Objectives:**

To assess the discrepancy between current classifications in the diagnoses of septate uterus and to correlate them with reproductive outcomes; to detect 3D transvaginal ultrasound (TVS) additional measurements, which can better correlate small indentation lengths >5 <10mm to reproductive failures.

**Materials and Methods:**

Observational study enrolling 664 women of reproductive age with 3D ultrasound diagnosis of an indentation length ≥3mm. For each patient a detailed reproductive history was taken before performing 3D transvaginal examination. Patients with previous uterine surgery or metroplasty were excluded.

**Main outcome measure(s):**

Indentation lengths >5 <10mm showed high discrepancy in the diagnosis of uterine septum between different classifications. For these small indentations additional 3D measurements (indentation angle, septal width and septal length/ fundal myometrial thickness (L/M) ratio) were correlated to infertility and recurrent miscarriage.

**Results:**

Among the cohort, 215 patients showed an indentation length >5 <10mm; 136 tried to conceive: 69 (51%) were infertile, 38 (28%) had recurrent miscarriages (≥2) and 5 (4%) had at least one delivery. Recurrent miscarriage significantly correlated to an indentation angle >134°; whereas infertility to an indentation width <32mm and a L/M ratio >75%.

**Conclusions:**

Wide discrepancies between different classifications are more evident in indentation lengths >5 <10mm. Additional measurements on 3D coronal section may help to evaluate the risk of infertility or recurrent miscarriage.

**What is new?:**

Additional 3D TVS measurements, beyond septal lengths, in particular for small fundal indentation, may help in predicting the risk of developing adverse reproductive outcomes.

## Introduction

Congenital uterine anomalies (CUAs) are deviations from normal anatomy due to embryological maldevelopment, fusion or reabsorption of Müller’s ducts or paramesonephs between the sixth and eighteenth week of gestation (Grimbizis et al., 2013). Lack of resorption or canalisation, as well as fusion defects lead to CUAs such as complete septate, partial septate or arcuate uterus.

Uteri with indentations of the fundal cavity, which has been defined as arcuate or subseptate, are the most common CUA and are often associated with reproductive problems (infertility, recurrent miscarriages, preterm delivery, foetal malpresentation and foetal growth restriction) (Grimbizis et al., 2001; Gergolet et al., 2012; Chan et al., 2011; Saravelos et al., 2008).

Simόn et al. (1991) reported the prevalence of Mullerian anomalies ranging from 0.6% to 38%. This wide variation may be due to the difference in classification systems and diagnostic modalities used.

The diagnosis of CUAs was previously made on hysteroscopy, laparoscopy, hysterosalpingography and magnetic resonance imaging (MRI). However, now three-dimensional (3D) transvaginal sonography (TVS) is considered the gold standard for their assessment as it is less invasive and can classify the various types of uterine anomalies correctly. (Kupesic, 2001; Salim et al., 2003; Ghi et al., 2009; Bermejo et al., 2010).

One of the most important issues regarding septate uterus is the lack of a universally accepted definition of the condition. American Fertility Society (AFS) is the most widely utilised system for classifying uterine anomalies: it includes septate uterus, based on subjective interpretation of anatomical type, using the coronal aspect of the uterus without any measurable criteria (The American fertility Society, 1988). Moreover, the scientific community introduced new classifications due to the variation seen with 3D TVS. The first purely ultrasound classification is the Salim classification (Salim et al., 2003) and is based on the observation of the fundic contour of the cavity, the measure of the fundic indentation and the angle created by this. According to Salim modified AFS classification, internal fundal indentation can be classified as arcuate or septate uterus if the angle is more or less 90° (Salim et al., 2003). In 2013, a consensus was introduced by a joint working group between the European Society of Human Reproduction and Embryology (ESHRE) and the European Society for Gynaecological Endoscopy (ESGE) under the common name of CONUTA (Congenital Uterine Anomalies) who eliminated the category of the arcuate uterus. (Grimbizis et al., 2013). This appears in line with the thinking of some authors who consider the arcuate uterus as a normal variant rather than an anatomical or developmental anomaly (Buttram and Gibbons 1979; Donnez 2018; Surrey et al., 2018). ESHRE/ESGE classification was hence revised and implemented by further detailed definitions in 2016. (Grimbizis et al., 2016)

Nonetheless, several authors have criticised the ESHRE/ESGE classification, insinuating that the adoption of this guideline inevitably led to an overestimation of the actual number of septate uterus and consequently to a greater number of surgical operations (Ludwin et al., 2019; Knez et al., 2018; Saridogan et al., 2020); this was despite ESHRE/ESGE not giving any treatment indications. In 2016, the American Society for Reproductive Medicine (ASRM) released a new classification of uterine malformations: this classification considered normal uterus with indentations up to 1cm and septa with indentations starting from 1.5 cm with an angle between endometrial layers <90°. This however posed further problems to the already complex classification of the uterine malformations, introducing the so-called “grey zone”, and opening further dilemmas for clinicians. (Practice Committee of the American Society for Reproductive Medicine, 2016). The more recent classification is the CUME (Congenital Uterine Malformation by Expert, 2018), classification proposed by 15 experts is also based on ultrasound criteria (measurements, angles and ratios) and was introduced in order to overcome the diagnostic difficulties generated by the previous classifications (Ludwin et al., 2018).

In 2021, ASRM proposed a new classification system, improving the AFS 1988 classification: authors considered normal or arcuate uteri with indentations ≤ 1 cm and an angle > 90° and classified as septate uteri with indentations > 1 cm and an angle < 90°. They also added to the previous classification of cervical, vaginal, and other complex anomalies. (Pfeifer et al., 2021)

All classifications of CUAs present strengths and weaknesses and have been criticised for different aspects. With regards to small indentations of the fundus, the question is still open for the real reproductive impact of the defect and the need for surgical treatment.

The main objective of the present study was to assess the discrepancy between the classifications currently in use (Salim/AFS 2003; ESHRE/ ESGE 2013-16; CUME 2018; ASRM 2021) in the diagnosis of small indentation lengths and to ultimately correlate this diagnosis with the reproductive outcomes.

A secondary aim was to detect further 3D TVS additional measurements in the group that showed the most discrepancy between the different classifications, the subseptate uteri, that may correlate with a negative reproductive outcome.

## Materials and Methods

### Setting and participants

In this retrospective observational study, we reassessed offline the uterine volumes of patients who showed a uterine cavity with internal fundal indentation of ≥ 3mm. All included patients underwent 2D, 3D and power Doppler TVS examination during the secretory phase (18 °-24° day) of the cycle as calculated based on the last menstrual period reported by the patients. In this study, we recruited patients less than 50 years of age whom had not yet reached menopause and without a record of on-going pregnancy. Other inclusion criteria were availability of an accurate reproductive history and optimal stored 3D volumes.

Exclusion criteria were complete septate uteri, previous metroplasty, myomectomy or other hysteroscopic surgery on the cervical canal or vagina, sub-optimal 3D images. Pregnancy (positive beta-HCG test), menopause, malignancy of the reproductive tract, benign endocavitary pathology (myoma, polyp, adenomyosis).

*Ethical approval:* Institutional review board approval was obtained (No. 119.21) and informed consent to data utilisation was signed by all patients.

### Clinical history and symptoms

Patient information was recorded according to a pre-established format using the File maker pro® software Version 9.0. The collected data included: the date of birth and age at the time of the ultrasound, body mass index (BMI), age of menarche, parity, menstrual cycle characteristics, last menstrual period, previous surgical interventions, endocrinological conditions, autoimmune pathologies or other childhood illnesses and familiarity for metabolic and oncological diseases.

Patients who tried to conceive were also asked about their reproductive history. In case of previous pregnancies women completed a questionnaire about their pregnancy, specifically conception (spontaneous or through assisted reproductive technologies (ART)), antenatal complications and the mode of delivery.

Reproductive complications were defined as follows: preterm birth before 37 completed weeks of gestation (Quinn et al., 2016), miscarriage as a loss of pregnancy during the first 23 weeks of gestation, recurrent miscarriage as a history of two or more loss of pregnancy during the first 23 weeks of gestation, infertility defined as attempted conception after one year (or longer) of unprotected intercourses (Practice Committee of the American Society for Reproductive Medicine, 2013) and ectopic pregnancy defined as any pregnancy implanted outside of the endometrial cavity (Hendriks et al., 2020).

### Ultrasound examination

The ultrasound examinations were performed using a Voluson E6 or E8 device (GE Healthcare, Zipf, Austria) with a transvaginal probe. The ultrasound settings were standardised and identical for all subjects. The scan was first involved with a conventional two-dimensional (2D) ultrasound assessment of the pelvis. The uterus, endometrium and adnexae were evaluated. The 2D examination was followed by acquisition of 3D data using the 3D volume mode. The 3D volume mode displayed a truncated sector that was adjusted to define the area of interest. The sweep angle was set to 120° to include the entire uterus and a 3D dataset was then acquired using the high-quality, slow-sweep mode. Furthermore, two to four static volumes of the uterus in grey scale were obtained from the transverse plane in order to obtain an optimal coronal view that visualised both uterine horns, or in some cases, from the sagittal plane. For each patient, several volume acquisitions were taken throughout the TVS examination in order to minimise the changes of the uterine cavity due to uterine contraction. Datasets of the uterus from each subject were stored on recordable digital video discs for subsequent analysis. Stored uterine ultrasound volumes were subsequently retrieved for offline analysis. The coronal view reconstruction technique was standardised according to the following criteria:

- on the multiplanar sectional view with straight or curved line (omni-view) along the endometrial stripe; VCI (volume contrast imaging) on multiplanar view at 2-4 mm slice thickness.
- on the volume with the rendering box adjusted in window A and B of the multiplanar view, to include all the uterine fundus and the green rendering line set from front to back, the green line straight or curved along the endometrial stripe, rendering mixed light surface and gradient light.

**Figure 1:**
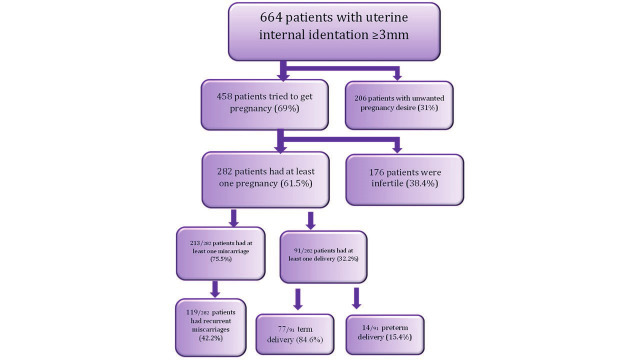
Reproductive history of the 664 patients with septate uterus at 3D transvaginal ultrasound included in this study.

Analysis of uterine architecture was carried out on a standardised coronal plane using the interstitial portions of the fallopian tubes as reference points and optimal visualisation of isthmic portion. The following specific measurements were determined (Figure 1):

- septal width (W): the distance between the two internal tubal ostia.
- uterine fundal wall thickness (M), the distance from interostial line and the external uterine serosa.
- fundal indentation angle (α), the angle between the two endometrial layers.
- the indentation length (L), the distance from the tip of the fundal indentation to the interostial line.

Complete septate uteri were excluded. Uteri with a fundal internal indentation of less than 3mm were also excluded from the study population.

Based on these measurements, all the uteri were classified as septate or arcuate/normal according to the following classifications: Salim (2003), ESHRE/ESGE (2013-16), CUME (2018), ASRM (2021). For each classification the uterus was defined at septate according to the following criteria:

Salim (2003): a uterus with convex external profile or an external fundic indentation depth <10 mm, which separate the uterus into hemi cavities and angle of internal indentation <90°.

ESHRE/ESGE: internal fundal/uterine indentation depth >50% of uterine-wall thickness and external indentation depth <50% of uterine- wall thickness, with uterine-wall thickness measured above interostial/intercornual line (L/ M>50%).

CUME 2018: internal fundal indentation depth ≥1 cm and external fundal indentation depth <1 cm or with angle of internal indentation <140° or with a ratio internal indentation/ myometrial thickness >110% (L/M).

ASRM 2021: internal fundal indentation depth >1cm cm, angle of internal indentation <90° and external fundal indentation depth <1 cm.

Further analysis was carried out in the group of subseptate uterus, which show more discrepancy between different classifications (small septa 5-10 mm) to correlate different ultrasound parameters with reproductive outcomes.

### Statistical analysis

All data collected were analysed with the statistics functions of EXCEL programme (Microsoft ® Excel® for Microsoft 365 MSO Version 2202 Build 16.0.14931.20128). Initially, a correlation between reproductive outcomes and 3D ultrasound parameters was performed. Comparison between the different classifications in the diagnosis of septate uterus was then evaluated. Further analysis was carried out in the group of subseptate uteri, which show more discrepancy between different classifications (small septa 5-10 mm) to correlate different ultrasound parameters with reproductive outcomes, excluding women with unwanted pregnancy. Of these subgroups, other 3D measurements of the uterine cavity were analysed (α, L, M, L/M) and correlated to the reproductive failures (infertility, recurrent miscarriages, preterm delivery).

Measurements concerning quantitative variables were developed with arithmetic mean and standard deviation (SD). Qualitative or categorical variables were expressed as number of cases (n) and relative percentage (%). The difference between quantitative variables was tested using the t-Student’s -test (2-tailed) while the Fisher’s test (2-tailed) was chosen to compare categorical or qualitative variables. Comparisons between both groups were performed using the technique of variance or ANOVA. A p value less than 0.05 was considered statistically significant. Cohen’s K was also calculated to verify the degree of accuracy and reliability of the classifications considered.

Descriptive statistics were first presented for all the variables analysed. Mann-Whitney tests were carried out on the quantitative variables to verify whether they have the same distribution in the two sub-categories of the dichotomous variables.

Where the test led to the rejection of the null hypothesis of the same distribution (i.e. where the p-value of the test is lower than the 0.05 threshold level) it was concluded that the distribution was significantly different in the two subgroups and a receiver operating characteristics (ROC) curve was used to obtain the optimal cut-off.

In the ROC curves, in addition to the optimal cut-off, sensitivity and specificity associated with the cut-off itself and the AUC (Area Under the Curve) were evaluated.

## Results

### Study population

Of the 753 patients, 664 with a 3D ultrasound diagnosis of a uterine internal fundal indentation of ≥3mm met our inclusion criteria for this study. They had a mean age of 34.6 years (range 16- 50). 458 (69%) tried to conceive and of these, 282 (61.5%) obtained at least one spontaneous pregnancy, while 176 (38,4%) had primary infertility. 119/282 (42%) patients had recurrent miscarriage. 91 patients had at least one delivery, however, 15.4% of these had a preterm delivery (Figure 1). Patients came to our attention reporting these main indications: suspected uterine anomalies (223 patients), infertility (106 patients), recurrent miscarriages (95 patients), endometriosis (51 patients) and other indications (189 patients). The remaining 89 patients were excluded for various reasons: 20 patients for suboptimal 3D images, 20 patients because of a benign pathology of the uterine cavity, 19 patients for an unclear medical history and 30 patients for other causes of infertility (endometriosis, tubal factors, male factors). Patients who had undergone previous metroplasty and patients in menopause were not considered in advance.

### Association between reproductive outcomes and 3D septal measurements in our study population

The correlation between reproductive outcomes of our population study with pregnancy desire (n = 458) and 3D septal measurements is reported in Table I. We observed that patients who delivered had a significant higher septal fundal indentation length (L) (8.5±5.4) compared to those with recurrent miscarriages (7.2±4.3) and primary infertility (7.1±4.9). Patients with infertility had a smaller fundal indentation width (W) (26.9 ± 5.6 mm) compared to those who delivered at term of pregnancy (29.9 ± 6.9 mm). Finally, Ratio L/M was significantly lower in patients with recurrent miscarriages (75.1± 55.3) compared to patients who delivered (100.0± 96.8).

**Table I:** Correlation between reproductive outcomes and 3D ultrasound parameters in patients trying to conceive.

Patients trying to get pregnant (N=458)	%			UTERINE SEPTAL MEASUREMENTS				
		Angle α mm mean± SD	Length (L) mm mean± SD	Septal Width (W) mm mean± SD	Myometrial thickness (M) mm mean± SD	Ratio L/M % mean± SD	Ratio W/L % mean± SD	Ratio L/α % mean± SD
Primary infertility (N=176)	38% (176/458)	127.3±23.7	7.1±4.9^b^	26.9±5.6^c^	9.3±2.3	83.8±70.3	26.3±17.2	7.1±9.6
At least one pregnancy (N=282)	62% (282/458)	123.4±25.5	7.5±5.1	27.5±5.9	9.5±2.6	92.1±91.4	27.4±17.4	8.0±11.0
Only one miscarriage (N=94)	33% (92/282)	119.1±29.0	8.2±5.3	29.1±5.7	9.3±2.6	101.8±101.4	27.9±17.9	9.3±11.5
Recurrent miscarriage (N=119)	42% (119/282)	125.6±22.3	7.2±4.3^a^	28.2±6.4	10.6±3.09	75.1±55.3^d^	25.8±14.4	6.9±6.9
Delivery (N=91)	32% (91/282)	121.1±26.5	8.5±5.4^ab^	29.8±6.8^c^	10.1±2.7	100.0±96.8^d^	28.3±16.9	8.9±9.7
Term delivery (N=77)	27% (77/282)	123.7±25.7	8.1±5.4^ab^	29.9±6.9^c^	10.3±2.8	95.7±98.7	26.8±16.8	8.4±9.9
Preterm delivery < 37 wks (N=14)	5% (14/282)	102.0±30.2	11.3±5.4^ab^	28.9±5.4	9.0±2.6	143.6±94.8	40.2±19.5	13.8±10.8

^a^ p<0.05 delivery vs recurrent miscarriage; ^b^p<0.05 delivery vs primary infertility; ^c^p<0.05 term delivery vs primary infertility; ^d^p<0.05 delivery vs recurrent miscarriage.

### Population study according to the different classifications for septate uteri

**Figure 2:**
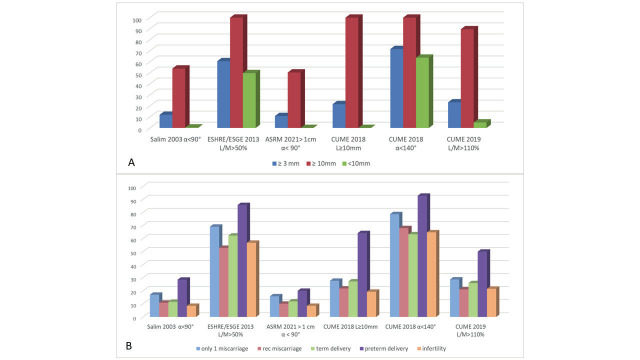
A) Comparison between different classifications in the diagnosis of septate uterus in the population with a fundal indentation ≥ 3 mm , ≥10 mm and <10 mm. B) Relation between reproductive outcome and diagnosis of subseptate uterus according to the different classifications. The percentage (%) of reproductive failure showed a high discrepancy between different classifications. For example, ESHRE-ESGE diagnosis of septate uterus was associated to 52.9% of recurrent miscarriages whereas in ASRM or in CUME (L≥10 mm) classification of septate uterus the percentage of recurrent miscarriages was lower (3.3 % and 21.8%).

All patients were classified as septate or arcuate/ normal uterus according to the different classifications. In our study population of 664 patients with internal fundal indentation ≥3 mm, septate uterus was classified according to Salim (2003) definition in 11.9%; 60.5% according to ESHRE/ESGE (2013-16) parameters; 10.8% for ASRM (2021) definition; for the 3 different CUME definitions in 21.5%, 71.5%, 23.2% respectively (Figure 2A). A high discrepancy was observed between ESHRE/ESGE and the other three classifications (ASRM, CUME, Salim) regarding the definition of septate uterus as also in the relationship observed between reproductive outcome and diagnosis of subseptate uterus (Figure 2B). This discrepancy was less when the septal fundal indentation length was more than 10 mm and was more evident in smaller indentation (Figure 2A). Thus, evaluating the group of small indentation lengths < 10 mm, we observed a particular higher discrepancy in the subgroup with an indentation length between 5 and 10 mm, whereas patients with indentation ≤5 and ≥10 mm showed similarity in type (Table II).

**Table II:** Comparison between different classifications in the diagnosis of subseptate uterus in our population, divided in three groups. Cohen’s K value is used to evaluate the agreement rate between classifications:

Group A (L ≤ 5mm) N=306		Group B (L <5 > 10 mm) N=215		Group C (L≥ 10 mm) N=143	
Classifications	K value	Classifica tions	K value	Classifications	K value
Salim vs ESHRE/ESGE	0,75	Salim vs ESHRE/ESGE	0,1	Salim vs ESHRE/ESGE	0,7
Salim vs ASRM 2021	1	Salim vs ASRM 2021	0,9	Salim vs ASRM 2021	1
Salim vs CUME 140°	0,65	Salim vs CUME 140°	0,1	Salim vs CUME 140°	0,7
Salim vs CUME 1 cm	1	Salim vs CUME 1 cm	0,9	Salim vs CUME 1 cm	0,7
Salim vs CUME 110%	1	Salim vs CUME 110%	0,8	Salim vs CUME 110%	0,8
ESHRE/ESGE vs ASRM 2021	0,6	ESHRE/ESGE vs ASRM 2021	0,1	ESHRE/ESGE vs ASRM 2021	0,5
ESHRE/ESGE vs CUME 140°	0,64	ESHRE/ESGE vs CUME 140°	0,8	ESHRE/ESGE vs CUME 140°	1
ESHRE/ESGE vs CUME 110%	0,75	ESHRE/ESGE vs CUME 110%	0,2	ESHRE/ESGE vs CUME 110%	0,9
ESHRE/ESGE vs CUME 1 cm	0,75	ESHRE/ESGE vs CUME 1 cm	0,1	ESHRE/ESGE vs CUME 1 cm	1
ASRM 2021 vs CUME 110%	0,94	ASRM 2021 vs CUME 110%	0,8	ASRM 2021 vs CUME 110%	0,4
ASRM 2021 vs CUME 140°	0,66	ASRM 2021 vs CUME 140°	0,09	ASRM 2021 vs CUME 140°	0,5
ASRM 2021 vs CUME 1cm	1	ASRM 2021 vs CUME 1cm	1	ASRM 2021 vs CUME 1cm	0,5

We focused our attention on this cohort of patients (indentation length between 5 and 10 mm) since in this subgroup high discrepancy was detected when using each classification.

### Small uterine septa (indentation>5 and <10 mm) populations results and ROC curves analysis

In our study, of the 664 patients, 215 showed a fundal indentation length >5 <10mm. Of these, 136 tried to conceive before our ultrasound examination: 69(51%) were infertile, 65(48%) had at least one miscarriage, 38(28%) had recurrent miscarriages and 5(4%) had a preterm delivery.

We tried to understand how all the 3D parameters measured, beside indentation length, could best correlate with the risk of developing an adverse reproductive event. Table III lists the descriptive statistics relating to the 3D ultrasound measurements, in patients with a primary unexplained infertility (n = 69) and recurrent miscarriage (n=38).

**Table III:** Correlation between 3D measurements and the two subgroups: infertility and recurrent miscarriages, in the cohort with fundal indentation length >5 <10 mm. ap<0.05.

	Infertility			Recurrent miscarriage		
	YES n= 69	NO n= 67		YES n= 38	NO n= 98	
Variables Septal measurements	Mean ± SD	Mean ± SD	P value	Mean ± SD	Mean ± SD	P value
Lenght (L)	6.78±0.96	6.99±1.10	0.346	6.71±1.01	6.95±1.04	0.157
Angle α	126.41±9.38	127.03±11.2	0.671	129.58±11.0	125.60±9.87	0.036^a^
Width (W)	27.70±4.82	29.93±5.38	0.009^a^	29.39±6.24	28.56±4.77	0.298
Myometrium (M)	9.16±2.31	10.85±2.75^a^	0.000^a^	10.71±3.09	9.71±2.44	0.106
Ratio L/M	78.82±22.49	69.16±22.8^a^	0.008^a^	68.17±22.9	76.34±22.8	0.059
Ratio W/L	25.09±5.07	23.97±4.9	0.383	23.71±5.4	24.85±4.8	0.195
Ratio L/α	5.42±1.06	5.59±1.2	0.789	5.23±1.05	1.21±0.12	0.064

**Figure 3:**
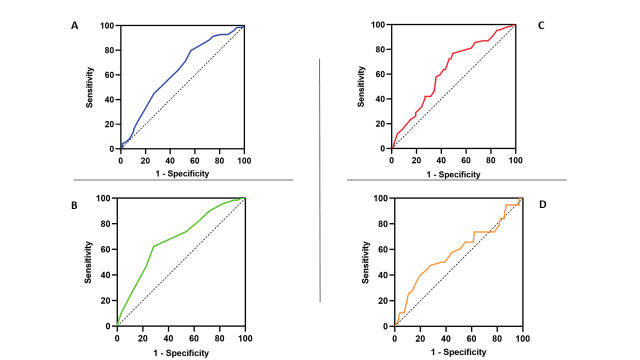
ROC CURVES. A) Width in infertile patients; B) Myometrium thickness in infertile patients; C) Ratio L/M in infertile patients; D) Angle α in patients with recurrent miscarriage.

Observing the test results, we noted that for three variables, width (W), myometrium thickness (M) and indentation length/myometrium thickness ratio (L/M), there were significantly different distributions in the infertile group compared to the non-infertile. Therefore, we proceeded to a ROC curve (Figure 3) to find the best cut-off for each of these parameters associated to infertility. The best cut-off identified in the ROC analysis for W was 31.5 mm (sensitivity 80%; specificity 57%, AUC 0.630; p =0.009). For M, the best cut off found was 9.4 mm (sensitivity 72%; specificity 80%; AUC 0.681; p = 0.0001) and for L/M ratio was 74.5% (sensitivity: 58%; specificity 65%; AUC 0.631; p=0.008). Therefore, patients with a small uterine fundal indentation (L=69mm) with W < 32 mm, M < 9mm and with L/M ratio > 75% had higher risk to be infertile.

Regarding the analysis on recurrent miscarriage, we observed only how the distribution of the angle between endometrial layers (α) appeared to be significantly different between the categories of patients with a history of recurrent miscarriage and without. We therefore proceed to make the ROC curve.

The best cut-off identified was 133.5° (sensitivity: 40%; specificity 20%; AUC 0.58; p = 0.036). Notably, patients with a small fundal indentation (6-9mm) and α more than 134° were more at risk for recurrent miscarriage. (Figure 1)

Concerning the results on preterm delivery and one miscarriage only, statistically significant differences in the distribution of the variables was not achieved. For this reason, the ROC curve was not indicated. It was concluded that the seven measures selected are non-discriminatory in determining the risk of preterm birth or having a single miscarriage.

## Discussion

In the recent years, since its introduction, 3D TVS has become the gold standard for the evaluation of mullerian anomalies. A coronal view of the uterus can be obtained along with accurate measurements of the septal length and fundal myometrium thickness (Grimbizis et al., 2013; Exacoustos et al., 2015; Graupera et al., 2015). Despite the measurements on 3D ultrasound being accurately defined and taken, classification of septate uterus is very different according to the most used classification systems. Therefore, in reproductive outcomes comparison, the first step is to define and diagnose the anomalies in the same way. For complete septum, all the classification systems agree in the diagnosis; however for subseptate uterus, especially for small septa, there is a great disagreement in papers, letters, and reviews. There is also discrepancy regarding the treatment of uterine sub septum, and if septa benefit from surgical treatment or not. (Alvero et al., 2021; Ludwin, 2020).

In this study, the focus is not to give treatment guidelines, but to create a path to understand the diagnostic and classification problems in the definition of subseptate uterus, with regards to reproductive outcome in untreated uteri. For this reason, we revaluate all the stored 3D volumes of uteri with internal indentation before any treatment; we reclassify them, according to the most recent definitions and correlated them to reproductive outcome in patients who try to conceive. We set a 3mm cut off to be sure to perform our evaluation of a real uterine indentation and our examination was done in the same menstrual cycle time in order to minimise the differences in the measurements during different phases of the cycle. We also took several uterine volume acquisitions to reduce the difference due to uterine contractions. (Van den Bosch et al., 2021)

With regards to other studies (Detti et al., 2021; Ludwin and Ludwin, 2015), we found significant differences in the diagnosis of subseptate uterus according to the recent classifications (Salim/AFS 2003, ESHRE/ESGE-2013-16 or CUME-2018, ASRM-2021) based on 3D TVS measurements. We also found a great difference in the percentage of reproductive outcome problems according to the definition of subseptate uterus. Despite our significant correlation observation between ultrasound measurements and recurrent miscarriage, preterm delivery or infertility, the percentage of these reproductive failures vary significantly according to the diagnosis of subseptate uterus when different classifications were considered.

The results of this study focused in small indentation lengths between 5-10 mm. In fact, for indentation length ≤5mm, the classifications appeared to have similar diagnoses and only in a small percentage according to ESHRE/ESGE were these uteri classified as septate. It should be considered that the ESGE/ESHRE classification (Grimbizis et al., 2013) never gave indication to treat these small anomalies. The L/M ratio gives some information on the type of anomalies, since low ratio correlates not only to reabsorption defect of the uterine septation but also to a fusion defect. On the other hand, a thin M is a contraindication to resection, especially in indentation ≤5 mm.

Therefore, we suggest that indentation ≤5 mm should be mentioned in the 3D report, but not classified as septate uterus. Most of the classifications are similar in the diagnosis of septate uterus with septal length ≥10mm. ASRM recently set the cut off at 1cm; it was previously defined in 2016 ≥1.5 cm. However, in the ASRM 2016 there was a grey zone, which failed to classify women with uterine internal indentation with depth between 1–1.5 cm. In the most recent ASRM version of 2021 a grey zone still remains in case of indentation length ≤ 1 cm and angle < 90° and length > 1 cm and angle > 90°.

The problem in classifications remains for smaller indentation between 5 and 10 mm, which are classified by ESHRE/ESGE mostly as anomalies (U2a 84%), whereas with other classifications as normal/arcuate. In this study, although limited by a small study group (indentation 5-10 mm) and bias of not always clear causes of infertility/ recurrent miscarriages, we observed a relative high percentage of these two reproductive failures. In this group of patients, we observed infertility when the indentation width was < 32 mm, the fundal myometrium was < 9 mm and the L/M ratio was > 75%. Furthermore, patients with a small indentation (5-10 mm) and an angle > 134° showed a higher risk of recurrent miscarriage compared to general population (28% vs 1-2%) (Duckitt and Qureshi, 2015). Looking at these results, we can hypothesise that small indentation, or septa for ESHRE classification, with a short width and thin fundal myometrium are more correlated to infertility due to a fusion defect and thin fibrotic septum. In contrast, large width (large angle) of the small indentation/ septum may permit an embryo implantation but results thereafter in miscarriage due probably to vascular or myometrial alterations of the septum. These results could be useful in the management and counselling of patients with small uterine indentation before they look for pregnancy, or before undertaking assisted reproductive technology (ART). The clinician can use these data to advise patients to undergo metroplasty or not, not only based on the age and the obstetric history, but also on the size of the indentation or septa morphology.

A limitation of this study is the absence of a control group with normal uterine cavity regarding reproductive failure. Unfortunately, our unit is dedicated to gynaecological pathologies that often cause reproductive problems (such as endometriosis, polycystic ovary) and so the percentage of infertility or miscarriage is high also in the case of normal uterine cavity. To to compare the frequency of negative reproductive outcomes, we referred to the general population reported in the literature. Further limitations reports state that reproductive problems often show multifactorial causes and the uterine morphology specifically in case of small uterine indentation could not always be the only cause of adverse outcomes.

In conclusion our results confirm high discrepancy between different classification systems, especially in small indentations. Furthermore this subgroup of patients appear to have a risk of infertility and recurrent miscarriage when correlated with other indentation measurements. The results may help the clinician to give better counselling to patients, especially in small fundal indentation, where current classification systems in use show different diagnostic results.

Further prospective randomised studies are needed to confirm our data and to better understand the impact of uterine cavity fundal indentation morphology (evaluated by several 3D ultrasound measurements and characteristic) on reproductive outcome.
